# Antibacterial synergistic behaviour of phytosynthesized magnesium oxide nanoparticles with clove essential oil

**DOI:** 10.1038/s41598-025-18967-y

**Published:** 2025-09-12

**Authors:** Asmaa O. Manaa, Hoda H. Baghdadi, Lamia A. Heikal, Lobna S. El-Hosseiny

**Affiliations:** 1 Department of Environmental Studies, Institute of Graduate Studies and Research, Alexandria University, Alexandria, 21526 Egypt; 2 Department of Pharmaceutics, Faculty of Pharmacy, Alexandria University, Alexandria, 21521 Egypt

**Keywords:** Green synthesis, MgO nanoparticles, Essential oils, Natural antimicrobials, Drug discovery, Microbiology, Nanoscience and technology

## Abstract

While researchers continue to search for new antibacterial agents, combination therapy as well as nanotechnology-based treatments allure as promising approaches to tackle antibiotic resistance. The present study aimed to phytosynthesize magnesium oxide NPs (MgO NPs and evaluate their potential antibacterial synergistic behaviour with different essential oils (EOs). MgO NPs phytosynthesized using thyme aqueous extract were quasi-spherical with an average size of 55.2 ± 12.8 nm and an elemental composition of 35.39% Mg and 51.07% O, as determined by SEM-EDX. FTIR elicited characteristic functional group peaks, while XRD confirmed their cubic crystal structure. The phytosynthesized MgO NPs and four EOs displayed variable antibacterial activity against *Staphylococcus aureus*,* Enterococcus faecalis*,* Escherichia coli*, and *Pseudomonas aeruginosa.* The checkerboard assay revealed that only clove and thyme EOs showed synergistic effects in combination with MgO NPs. Notably, MgO NPs-clove EO combination caused significant bacterial membrane damage as compared to their single counterparts in both *S. aureus* and *E. coli*. Moreover, oxidative stress was induced, as observed by the significant increase in the antioxidant enzyme activities (superoxide dismutase and catalase). Conclusively, the present results provide insights into the promising compounding of green-synthesized MgO NPs and clove EO as a bio-efficacious and eco-friendly approach to curtail antibiotic resistance.

## Introduction

The increasing prevalence of antibiotic resistance is one of the top global health jeopardies. The misuse of antibiotics in humans, animals and agriculture has created a state of silent pandemic which is expected to surpass any other cause of mortality by 2050. Moreover, the current arsenal of traditional antibiotic therapies is on the verge of failure in tackling such a challenge^1^. The combat of antibiotic resistance encompasses three pivotal strategies comprising surveillance, stewardship programs as well as research and development of new antibiotic therapies^2^. In this context, natural products and/or the development of nanotechnology-driven strategies have gained special interest due to their potential in addressing limitations of traditional antimicrobial therapies^3^.

Among the diverse array of nanomaterials, metal and metal oxide nanoparticles (NPs) have found their way across various fields, including biosensing, tissue engineering, catalysis, food packaging and biomedicine, owing to their distinctive physicochemical properties, such as large surface area, mechanical strength, photocatalytic activity and unique crystal morphology^4–6^. Metal/metal oxide NPs are synthesized by physical, chemical or biological methods. Physical approaches require a large amount of energy^4,7^. Meanwhile, the inclusion of hazardous chemicals in the chemical synthesis of metal/metal oxide NPs increases their toxicity and hazards to the environment and limits their use in the biomedical context. On the contrary, green approaches appeal as environmentally friendly, cost-effective, and sustainable methods for the synthesis of metal/metal oxide NPs^8^. Several metal/metal oxide NPs, including gold (Au), silver (Ag), iron (Fe), palladium (Pd), zinc oxide (ZnO) and copper oxide (CuO), have been synthesized via green methods using plant extracts, with enhanced biomimetic and biological properties. For instance, Au NPs have been synthesized from *Helianthus annuus* flowers and demonstrated notable antimicrobial activity^9^. Green-synthesized Ag NPs have also been extensively studied. For example, Ag NPs synthesized using *Tabebuia rosea* seed extract showed antibacterial, antioxidant, and antiproliferative effects^10^. In another study, Ag NPs prepared via *Justica wynaadensis* have exhibited strong antibacterial effects along with antioxidant, anticancer, anti-diabetic and anti-inflammatory properties^11^. Moreover, green-synthesized Fe and Pd NPs have demonstrated enhanced catalytic activity^12^, while ZnO and CuO NPs phytosynthesized using *Ulmus davidiana* aqueous bark extract have shown promising photocatalytic activity for environmental applications^13^. These findings reinforce the preference for green synthesis methods in metal/metal oxide NPs fabrication.

Nevertheless, metal/metal oxide NPs’ poor biocompatibility, toxicity, and contribution to the nano waste burden are obstacles that hamper their commercial prospects in nanomedicines. Amongst the various metal oxide NPs, Magnesium oxide (MgO) NPs have exceptional biocompatibility, non-toxic nature and robust stability in abrupt conditions. Besides, MgO is considered safe for human consumption according to the US Food and Drug Administration^6,14^, which puts it forth as an emerging candidate for tackling microbial infections^4,15,16^.

Plant-derived products are promising candidates as sources of antimicrobial agents that have been reported to counteract antimicrobial-resistant pathogens. Amongst plant-derived products, EOs captivated special attention owing to their diverse phytoconstituents and immense ethnopharmacological properties^17,18^. Their reported antimicrobial activity has encouraged researchers to investigate their synergistic potential with other EOs, plant extracts, or conventional antibiotics^18^. Notably, combining different EOs has been shown to exhibit synergistic antibacterial and antioxidant effects, making them promising candidates for multifunctional applications^19^. However, few studies addressed the synergistic potential of EOs with metal oxide NPs^18^; in particular, MgO NPs synergy has not been investigated to date.

To this end, the present study aimed to exploit natural products and green nanotechnology to offer insights into novel strategies for addressing pathogenic bacteria. Although the biosynthesis of MgO NPs using plant extracts has been widely studied, the use of thyme aqueous extract in this context is being reported for the first time. The phytosynthesized MgO NPs were then characterized and assessed for their antibacterial activity. Moreover, to the best of our knowledge, the current study is the first to investigate the antibacterial efficacy of MgO NPs in combination with EOs, including clove, rosemary, sage and thyme, aiming to explore potential synergistic interactions. Additionally, the potential underlying mechanism of action of MgO NPs, clove EO, and their combination on bacterial cell membrane integrity and oxidative stress induction was investigated.

## Results and discussion

### Visual appearance of the phytosynthesized MgO NPs

**Fig. 1:**
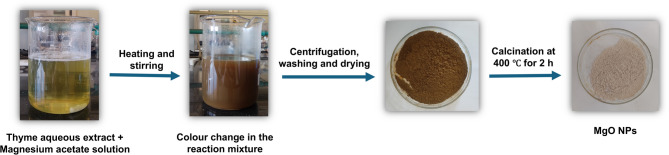
Schematic representation of *Thymus vulgaris* leaf aqueous extract-mediated synthesis of MgO NPs.

**Fig. 2:**
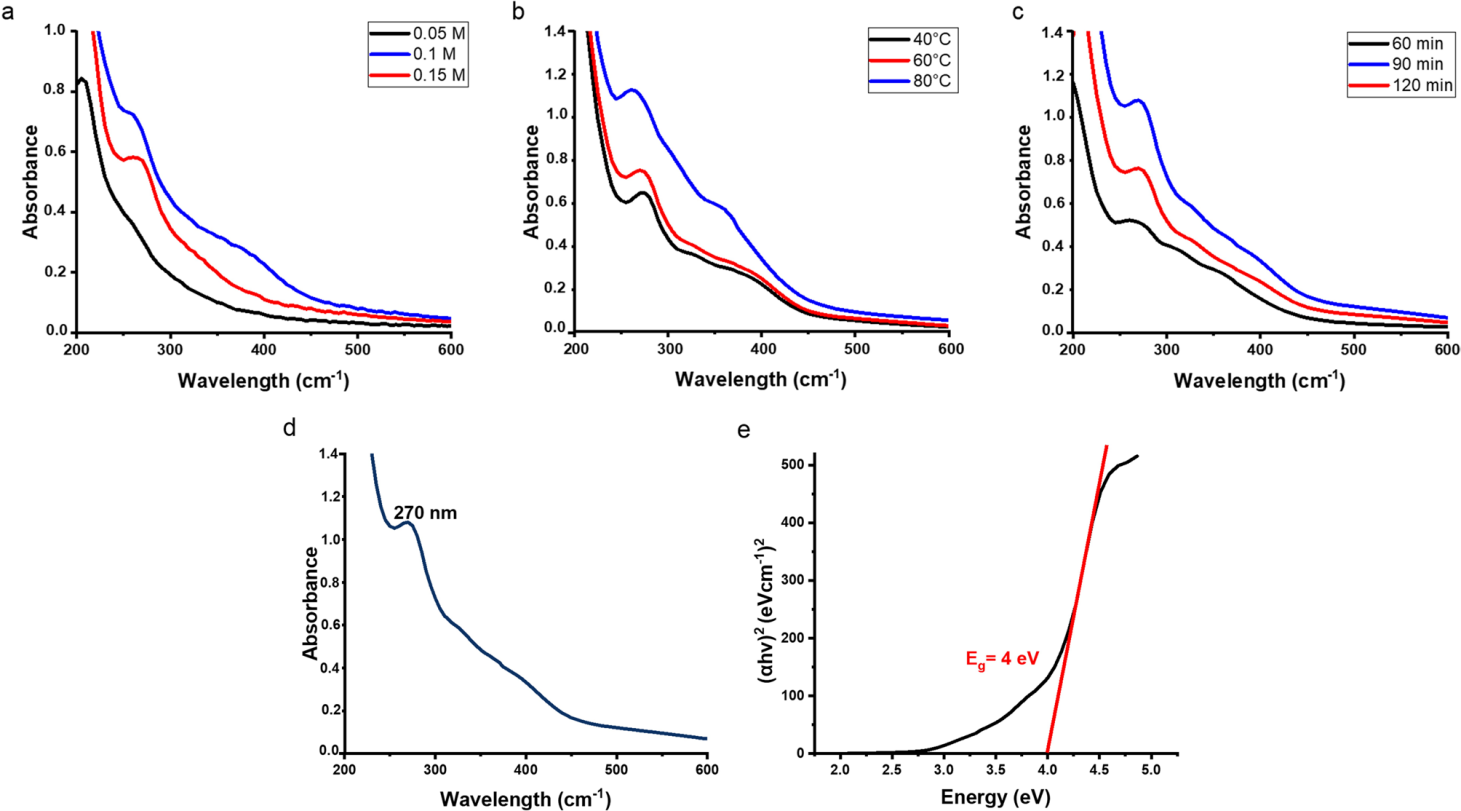
UV-vis spectrum of MgO NPs formed with different (**a**) precursor concentrations, (**b**) different temperatures, and (**c**) different reaction times. (**d**) UV–vis spectrum and (**e**) Tauc plot of MgO NPs phytosynthesized using thyme aqueous extract and 0.1 M precursor concentration and heated at 80 °C for 90 min.

The preliminary indication of MgO NPs phytosynthesis was observed by the colour transition from pale yellow to dark brown during the course of the reaction (Fig. 1), which was further confirmed by UV-Vis spectroscopy (Fig. 2). The observed colour alteration is indicative of the bio-reduction of magnesium salt via the phytochemicals present in the thyme extract^20^ and is ascribed to the collective oscillation of surface electrons at the nanoparticle interface, a phenomenon known as surface plasmon resonance^21.^

### Optimization of phytosynthesis parameters

#### Effect of precursor concentration

To scrutinize the influence of precursor concentration on the phytosynthesis of MgO NPs, three precursor concentrations (0.05 M, 0.1 M, and 0.15 M) were tested. As displayed in Fig. 2a, the highest absorbance peak was noticed at 0.1 M precursor concentration, indicating the highest nanoparticle yield^21,22^. In agreement with the present study, numerous previous studies used the same precursor concentration^23–26^.

Increasing precursor concentration from 0.05 M to 0.1 M enhanced nanoparticle yield. However, a further increase to 0.15 M has led to reduced absorbance. This reduction could be attributed to the limited ability of phytochemicals in the extract to fully reduce the numerous precursor molecules present in the reaction medium. Furthermore, higher precursor concentrations might lead to agglomeration of the NPs on the unreacted salt molecules, creating large particles. This aggregation could explain the weaker characteristic absorbance peaks of metal oxide NPs at higher precursor concentrations^21,22,26^.

#### Effect of temperature

In the present study, the magnesium precursor was heated with the thyme extract at three different temperatures, including 40 °C, 60 °C, and 80 °C, to assess the impact of reaction temperature on the phytosynthesis of MgO NPs. UV–vis spectroscopy results revealed that raising the temperature increased the absorbance, indicating higher nanoparticle yield, as shown in Fig. 2b.

Previous studies investigating the effect of temperature on metal NP synthesis have reported that both particle size and yield are directly proportional to the reaction temperature^27,28^, which is in agreement with the current results. This could be attributed to the inability of low temperatures to provide sufficient activation energy needed for the nucleation process and particle formation^22^. On the other hand, high temperatures accelerate the nucleation process and enhance NPs crystallinity^28^.

Although increasing the temperature to 80 °C enhanced the yield in the present study, the impact of higher temperatures was not further evaluated because phyto-mediated synthesis of metal NPs depends on the stability of the plant-derived phytochemicals^22,26,28^. Hence, high temperatures are unfavourable as they may lead to the degradation of these reducing phytochemicals^28,29^. Furthermore, previous studies have reported that increasing the reaction temperature above 60 °C decreased the yield of MgO NPs^21,22^.

#### Effect of heating time

The effect of reaction time on the MgO NPs synthesis was investigated by allowing the reaction to proceed for 60 min, 90 min and 120 min at a constant temperature of 80 °C. As shown in Fig. 2c 90 min was identified as the optimal time for higher NP yield as depicted by the maximum absorbance peak at 270 nm. Generally, extending the reaction time promotes the complete reduction of metal precursors, thereby improving the NPs yield. Nevertheless, excessively long reaction time may promote particle agglomeration and growth^22,28,30^.

In summary, the optimal reaction conditions for the phytosynthesis of MgO NPs in this study were a precursor concentration of 0.1 M, a temperature of 80 °C, and a reaction time of 90 min.

### Characterization of the phytosynthesized MgO NPs

#### UV-vis spectroscopy

UV–vis spectroscopy was employed for the optical characterization of the phytosynthesized MgO NPs^22,31^. UV-visible spectroscopic analysis of the optimized MgO NPs displayed an absorbance peak at 275 nm (Fig. 2d), which lies within the reported characteristic absorbance band range (260–280 nm) for MgO NPs^21,23,26^. The broad spectral band between ~ 300–450 nm is likely due to nanoparticles aggregation, which is known to cause spectral broadening, red shifts, and shoulders in UV–Vis spectra of metal/metal oxide NPs^21,77^.

A key attribute of metal oxide NPs is their bandgap energy, defined as it is the energy difference between the valence band and the conduction band. The direct optical bandgap can be calculated from the optical absorption spectra using the following equation:

$$\:{\left(ahv\right)}^{2}=A(hv-{E}_{g})$$

Where, α-absorption coefficient; *h*- Planck’s constant, *ν*-absorption frequency, A-constant of proportionality, and *E*_*g*_ is the energy band gap^21^. To determine the energy bandgap (*E*_*g*_) of the phytosynthesized MgO NPs, a plot of (*αhν*)^2^ versus photon energy (*hν*) was constructed, then the linear section of the plot was extrapolated to the photon energy axis. In the current study, the calculated energy bandgap of the phytosynthesized MgO NPs was 4 eV (Fig. 2e), which is in close agreement with earlier studies on green-synthesized MgO NPs^21,26^.

#### Scanning electron microscopy (SEM)

**Fig. 3:**
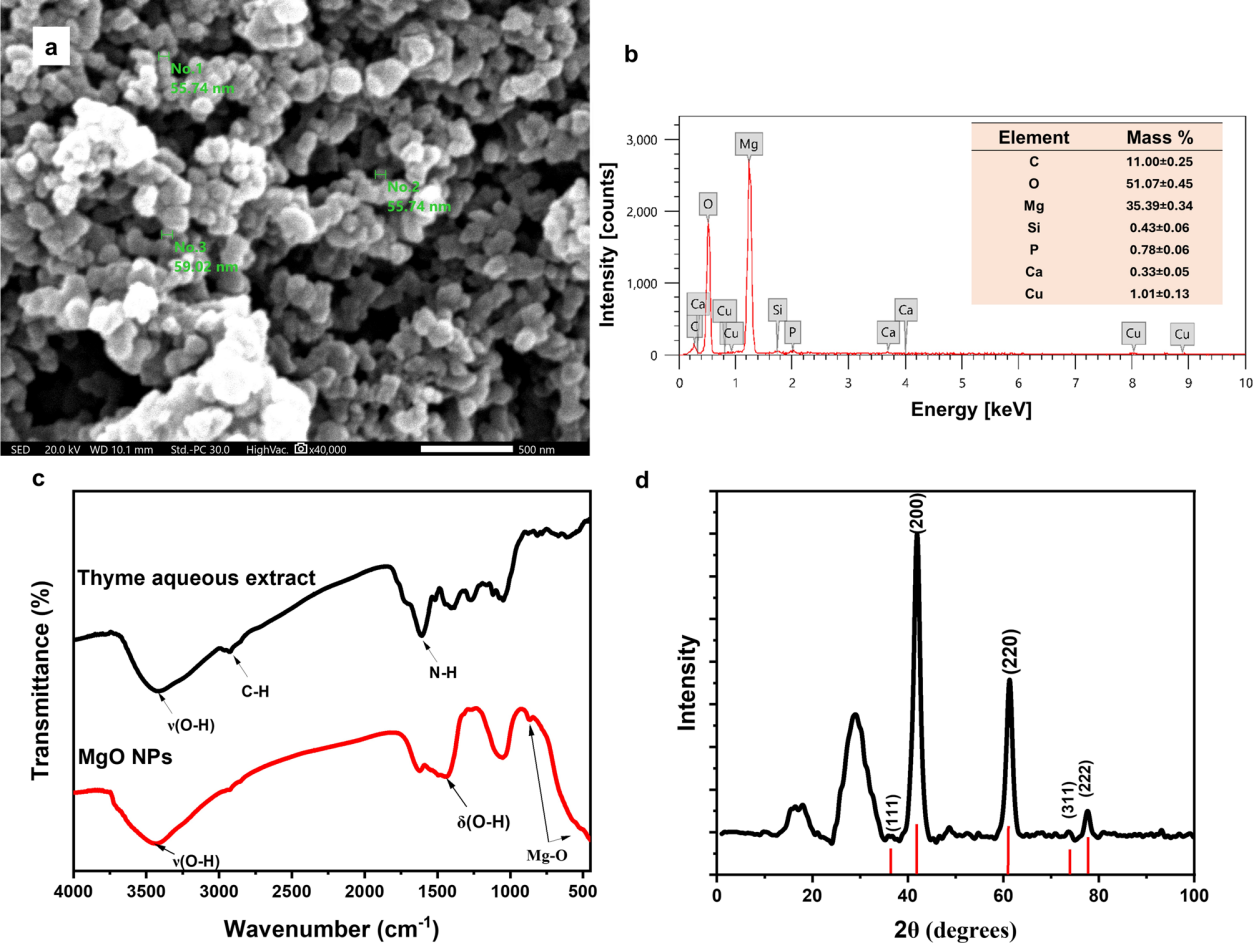
(**a**) SEM image of the phytosynthesized MgO NPs at magnification x40000, (**b**) EDX profile of the phytosynthesized MgO NPs, (**c**) FTIR spectra of *T. vulgaris* leaf aqueous extract and the phytosynthesized MgO NPs and (**d**) X-Ray diffractogram of the phytosynthesized MgO NPs.

The morphology and size of the phytosynthesized MgO NPs observed under SEM are shown in Fig. 3a, which demonstrating dense, nearly spherical particles with an average particle size of 55.2 ± 12.8 nm. The particles also appeared slightly agglomerated, which is probably attributed to the interactions and Vander Waals forces between the MgO NPs^32^.

The phytosynthesis of MgO NPs was further verified using energy-dispersive X-ray (EDX) analysis. As depicted in Fig. 3b, the EDX spectrum displayed strong magnesium and oxygen peaks with elemental compositions of 35.39% and 51.07%, respectively. An elemental carbon peak (11%) was also detected, which is probably ascribed to the presence of phytocomponents in the thyme extract, which acted as reducing and capping agents during the synthesis of MgO NPs. Throughout the phyto-reduction of the magnesium precursor during the green synthesis of MgO NPs, phytochemicals such as flavonoids, polyphenols, interact and contribute to the co-precipitation of Mg(OH)_2_ NPs. Subsequently, upon the calcination process, Mg(OH)_2_ NPs are converted into MgO NPs, residual phytocomponents are partially decomposed by heating, yet another part may deposit on the surface of the formed MgO NPs^33^. Overall, the present EDX analysis results qualitatively and quantitatively align with previous studies involving phyto-mediated MgO NPs synthesis^23,25,33^.

#### Fourier transform infra-red (FTIR)

Functional groups of the potential biomolecules in the thyme extract that may participate the bio-reduction and capping of the phytosynthesized MgO NPs, as well as probable chemical modifications due to the formation of MgO NPs, were investigated using FTIR spectroscopy. As shown in the FTIR spectrum of the thyme extract (Fig. 3c), a broad band was observed at around 3434 cm^− 1^, which is due to the asymmetric stretching vibration of aromatic O–H, indicating the presence of diverse bioactive compounds such as polyphenols, flavonoids, terpenoids, amongst others. The peak at 2927 cm^− 1^ was assigned to C–H stretching vibrations in the CH_2_ group (alkanes) present in the phytochemicals^23^. The band at 1616 cm^− 1^ corresponded to the N-H bending of the primary amine^34^. Moreover, the peak at 1385 cm^− 1^ is associated with C–H bending vibrations of aromatic amines^35^. The peak absorbed at ~ 1050 cm^− 1^ indicated the C-O stretching vibrations, which are common in alcohols or ethers that might be potentially present in thyme extract^20,36^.

In the FTIR spectrum of the phytosynthesized MgO NPs, the characteristic aromatic O-H broad peak at 3421 cm^− 1^ was also observed, implicating the involvement of the hydroxyl group in the thyme extract in the capping of the phytosynthesized NPs^23^. Nevertheless, the absence of the 2927 cm^− 1^ peak in the FTIR spectra of MgO NPs suggests the degradation of alkanes during the phytosynthesis process of MgO NPs^37^. The peak at 1449 cm^− 1^ corresponds to the bending vibration of the surface hydroxyl group (O–H) and physically adsorbed water molecules owing to the hygroscopic nature of MgO NPs^24,38^. The similarities between the FTIR spectra of the thyme aqueous extract and the MgO NPs suggest that many of the phytocomponents remain adsorbed onto the MgO NPs’ surface. Furthermore, the presence of peaks between 800 and 400 cm^− 1^ confirm the formation of MgO NPs and corresponds to the different vibration modes of the Mg-O bond. These findings are consistentwith the previously reported FTIR ranges for of various phytosynthesized MgO NPs^21,38,39^. The variations and shifts in the Mg–O vibrations reported in earlier studies could be ascribed to the interactions with the functional groups from different plant extracts or the utilization of different magnesium precursors^22^.

#### X-ray diffraction (XRD)

The XRD analysis was conducted to evaluate the size, purity, and crystallinity of the phytosynthesized MgO NPs. As seen in Fig. 3d, the diffractogram showed characteristic peaks of MgO NPs at 2θ of 36.86°, 42.04°, 62.03°, 74.15°, and 78.04° indexed to the crystal planes (111), (200), (220), (311), and (222), respectively. The most intense peak observed at 2θ = 42.04°, corresponding to the (200) plane, exhibited a d-spacing of 2.14 Å. Angular positions and intensity are well matched with the standard reference profile for the cubic phase of MgO NPs (JCPDS file no. 89-7746). Additional peaks at 2θ of 18° and 29.27° are likely due to graphite-like carbon impurities, which could possibly result from incomplete combustion of organic materials or carbonaceous residues from the plant extract, that is supported by the presence of carbon in EDX results. The resulting XRD pattern is in agreement with previously reported XRD profiles of phytosynthesized MgO NPs^20,40^.

The average crystallite size of the phytosynthesized MgO NPs was estimated by using the Debye–Scherrer equation:

$$D = ~\frac{{k\lambda }}{{\beta cos\vartheta }}$$

where *D* = particle size of the crystal, *k* = Scherrer constant (0.9), *λ* = wavelength of X-ray radiation (1.5406 Å), *θ =* the Bragg’s angle and *ϐ*= full width at half-maximum (FWHM) of the diffraction peak (2*θ =* 42.04°). The currently synthesized MgO NPs were found to have an average crystallite size of 50.4 nm, which is consistent with the crystalline size range seen under the SEM.

### Antibacterial activity

#### Antibacterial susceptibility testing

As shown in Table 1, both the phytosynthesized MgO NPs and the four investigated EOs exhibited considerable antibacterial activity against *S. aureus*,* E. faecalis*,* E. coli* and *P. aeruginosa* when assessed by the microplate-based resazaurin method. Albeit the immensity of factors that influence the antibacterial potency of MgO NPs and EOs, the present minimum inhibitory concentration (MIC) values demonstrated proximity with previously reported studies^41–44^. Upon examining the antibacterial effect of MgO NPs (25 nm) against two gram-negative and three gram-positive bacteria, the MIC of MgO NPs was 1 mg/mL for *E. coli* or *P. aeruginosa* versus 0.5, 0.7, and 1 mg/mL for *S. epidermidis*, *S. aureus*, and methicillin-resistant *S. aureus*, respectively^42^. In another study the MIC of MgO NPs (10–30 nm) prepared by the co-precipitation method was 0.5 mg/mL and 1 mg/mL against *E. coli* and *S. aureus*, respectively^43^. On the contrary, it was demonstrated that commercial MgO NPs of particle size 10–30 nm exhibited similar activities against *E. coli* or *S. aureus* with an MIC value of 2 mg/mL^41^. Moreover, in a study, which investigated the antibacterial potential of MgO NPs against *E. faecalis*, it was found that MgO nanospheres exhibited significantly higher antibacterial activity (MIC 1.17 mg/mL) than MgO nanorods (MIC 18.75 mg/mL)^44^.

**Table 1:** Minimum inhibitory concentrations (MICs) (mg/mL) of MgO NP, clove EO, thyme EO, Rosemary EO and Sage EO on selected microorganisms determined by microdilution method.

	MIC (mg/mL)					
	MgO NPs	Clove EO		Thyme EO	Sage EO	Rosemary EO
*S. aureus*	0.75	0.625		0.625	5	2.5
*E. faecalis*	1.5	0.625		0.625	5	2.5
*E. coli*	3	0.625		0.625	5	2.5
*P. aeruginosa*	6	0.625		0.625	5	5

Several factors were reported to influence the antibacterial activity of metal oxide NPs^42,45^. For instance, it has been proposed that decreasing particle size of NPs increases their antibacterial potency^46^. The underlying rationalization of this inverse relationship is that the decrease in particle size leads to an increase in the effective surface area^45^. Beyond this consensus, the shape and method of preparation of metal oxide NPs are also reported to influence their antibacterial properties. Metal oxide NPs of complex morphologies exhibit different mechanisms than other simple-shaped counterparts. For example, ZnO nanopyramids were reported to be more effective in inhibiting bacterial growth than other shapes, and it was concluded that metal oxide NPs with multiple edges or vertices are bequeathed with exceptional binding and conformational frustration of bacterial enzymes^47^.

Several methods have been employed for the preparation of metal oxide NPs, including physical, chemical and biological methods^28^. These methods undoubtedly affect the surface chemistry, shape and morphology of the synthesized NPs. Moreover, the use of capping agents, surfactants and stabilizing agents impart variability in NPs characteristics, which consequently influence the activity of the fabricated metal oxide NPs^48^. In a comparative study of the antibacterial activity of green and chemically synthesized MgO NPs, it was reported that, compared to chemically synthesized NPs, green-synthesized NPs exhibited a higher antibacterial effect against the tested gram-positive and gram-negative bacteria^49^. Consequently, when making comparisons concerning the potency of metal oxide NPs, it should be noted that NPs’ properties affect their antibacterial activity^45^.

There exists a discrepancy in the literature regarding the potency of metal oxide NPs’ activity against gram-positive bacteria and gram-negative bacteria^50–53^. As per the MIC results in the current study, the phytosynthesized MgO NPs exhibited higher potency against the tested *S. aureus* compared to the gram-negative *E. coli.* The structural difference in the cell wall of gram-positive and gram-negative bacteria may account for their varying sensitivities. In this regard, it has been postulated that the cell wall of gram-positive bacteria is composed of a thick peptidoglycan layer and teichoic acid that can be easily crossed by metal oxide NPs compared to the gram-negative cell wall, which is complex and contains an outer membrane of lipopolysaccharides^54^.

#### Synergistic potential of the phytosynthesized MgO NPs and EOs

The escalating antimicrobial resistance has become an alarming global health threat. Combination therapy is an alluring avenue of research to tackle this challenge^55^. Numerous studies have reported the antimicrobial potential of both EOs and metal oxide NPs, nevertheless the combination between metal oxide NPs and EOs is so far limited. In the current study, the potential synergistic effect of MgO NPs and four EOs has been investigated by the checkerboard method. As demonstrated by the FICI results (Table 2), the combination of clove, thyme, rosemary and sage EOs with MgO NPs resulted in synergistic, additive, or even indifferent effects. Both clove and thyme EOs showed synergy with MgO NPs against *S. aureus*, *E. faecalis*, and *E. coli* versus an additive effect against *P. aeruginosa*, whereas rosemary and sage elicited additive and indifferent effects, respectively.

**Table 2:** FIC index for the combined activity of MgO NPs with different EOs against selected microorganisms. Ind, Add and Syn indicate indifference, additive effect and synergy, respectively.

	MgO NPs + Clove EO	MgO NPs + Thyme EO	MgO NPs + Rosemary EO	MgO NPs + Sage EO
*S. aureus*	0.5 (Syn)	0.5 (Syn)	1 (Add)	1.25 (Ind)
*E. faecalis*	0.5 (Syn)	0.5 (Syn)	1 (Add)	1.25 (Ind)
*E. coli*	0.5 (Syn)	0.5 (Syn)	1 (Add)	1.25 (Ind)
*P. aeruginosa*	1 (Add)	1 (Add)	1.25 (Ind)	1.25 (Ind)

Essential oils are plant-derived agents that have a complex chemical composition. They are composed of a mixture of terpenes and terpenoids, which bestow them with inherent synergy as compared to their solitary components^56^. It can be depicted from the present results that the chemical composition of EOs affected the outcome of their association with MgO NPs. Phenolic terpenes comprising thymol and eugenol have been reported to be the major constituents in thyme and clove EOs, respectively^57,58^, whereas rosemary EO is constituted mainly of the monoterpene ether “cineole” and sage EO is reported to be constituted of both ether and ketonic monoterpenes comprising cineole and thujone^59,60^. Accumulating evidence has demonstrated that EOs constituted of oxygenated monoterpenes of phenolic or aldehydic nature exhibit substantially higher antibacterial activity versus those constituted of ketonic, alcoholic, or ethereal terpenoids^61,62^. Conceivably, it can be inferred that the variability in the major component of the tested EO may account for the outcome of the combination of the investigated EOs with MgO NPs. Both thyme and clove EOs, constituted by phenolic terpenoids, exhibited synergy in combination with MgO NPs as compared to either rosemary or sage EOs, which exhibited additive and indifferent antibacterial effects against *S. aureus* and *E. coli*.

The combination of metal/metal oxide NPs and EOs revealed variable outcomes ranging from decreased antibacterial activity to a significant increase in antibacterial effect and a shift in the spectrum of activity^63^. For instance, Ag NPs showed a synergistic antibacterial effect with EOs of *Kelussia odoratissima* and *Teucrium polium*^64^. Ag NPs also displayed synergistic antimicrobial activity against skin pathogens when combined with *Acanthospermum australe* EO^65^. Similarly, Ag NPs combined with eucalyptus EO exhibited a synergistic effect on the growth of *E. coli*, *S. enterica*, and *B. subtilis*^66^ and *Nigella sativa* EO coated with Au NPs effectively controlled the growth and biofilm formation of *S. aureus*^67^. Furthermore, rosemary and oregano EOs with Ag and ZnO NPs incorporated into pullulan films showed enhanced antibacterial effectiveness against *S. aureus*, *L. monocytogenes*, *E. coli*, and *S. typhimurium*^68^.

In the present study, while both clove and thyme EOs demonstrated similar MIC values and synergistic effects with MgO NPs, CEO was considered for further investigation owing to its prevalent use in traditional medicine and its potential for broader practical applications. Moreover, its prominent eugenol content, combined with MgO NPs, offers a prospect to create a formulation with enhanced antimicrobial effects, which may have a promising biomedical perspective^69^. Moreover, since *S. aureus* and *E. coli* displayed higher susceptibility towards MgO NPs than *E. faecalis*, and *P. aeruginosa*,* S. aureus* and *E. coli* were considered for further investigation of the potential antibacterial mechanism as typical representatives for gram-positive and gram-negative bacteria,.

#### Cell membrane integrity

The antibacterial mechanisms of metal oxide NPs have been investigated in several studies; however, there is no consensus on their exact antibacterial mechanism. Moreover, MgO NPs received marginal attention as compared to other metal oxide NPs, including zinc oxide, ferric oxide and titanium oxide NPs. Proposed mechanisms of metal oxide NPs embrace lipid peroxidation, cell membrane damage, enzyme inhibition and proteolysis^46,50,53^. In the present study, to depict the potential antibacterial mechanism of the phytosynthesized MgO NPs, clove EO and their combination, cellular membrane integrity was evaluated by assessing LDH activity as an index for cell membrane disruption as well as the leakage of nucleic acids and proteins.

**Fig. 4:**
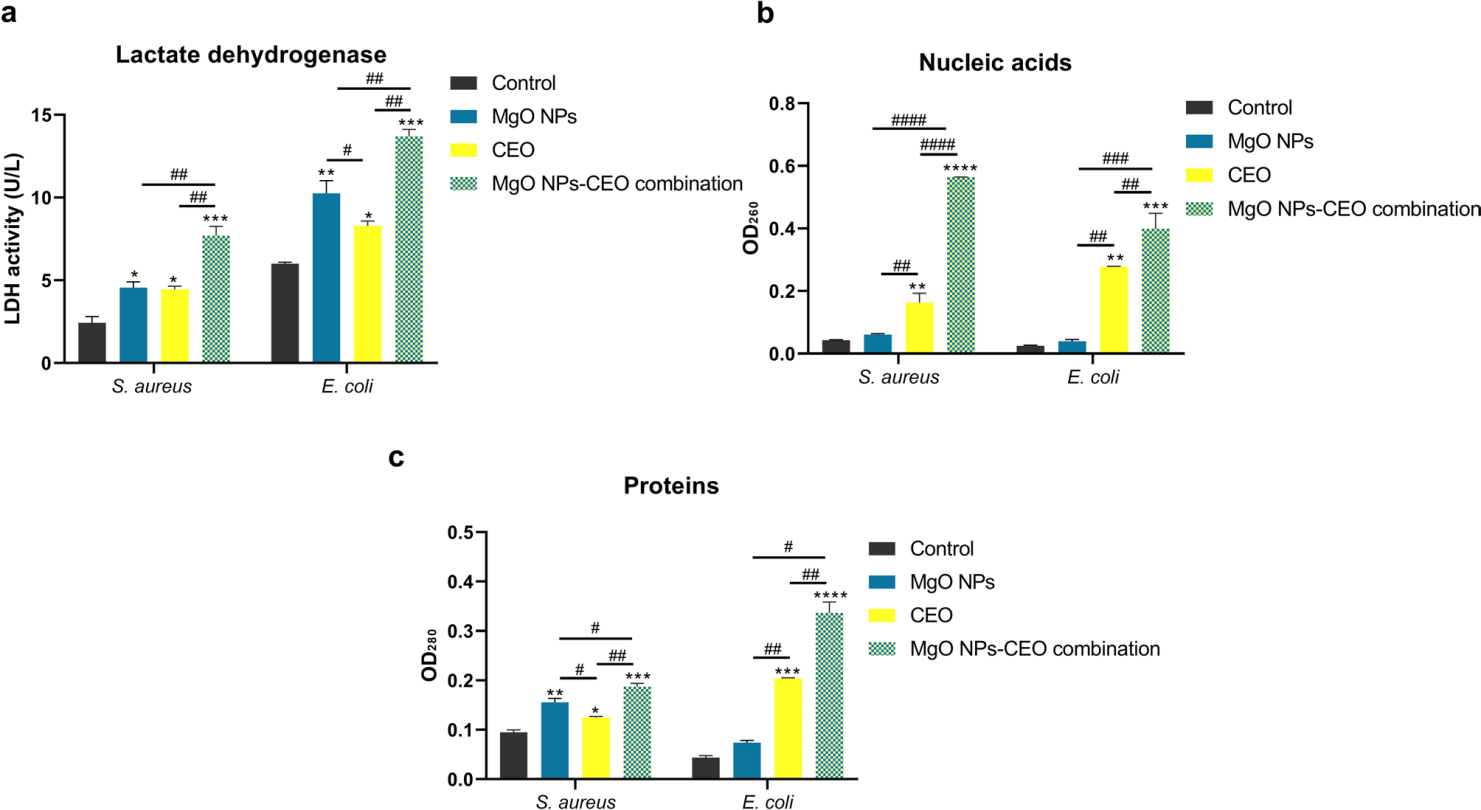
Effect of treatment with 1MIC of the phytosynthesized MgO NPs, clove essential oil (CEO) and MgO NPs-CEO combination on (**a**) lactate dehydrogenase (LDH) activity, leakage of (**a**) nucleic acids and (**b**) proteins in *S. aureus* (ATCC 29213) and *E. coli* (ATCC 35218). Data are presented as mean ± SD (*n* = 3), and statistical significance was determined by one-way ANOVA test. * designates a significant difference compared to control, whereas # designates a significant difference relative to the specified groups (*/#*p* < 0.05, **/##*p* < 0.01, ***/###*p* < 0.001 and ****/####*p* < 0.0001).

As demonstrated in Fig. 4a, MgO NPs alone and in combination with CEO significantly increased LDH activity. Reported as an intracytoplasmic enzyme, the increase in LDH level is attributed to its leakage as a result of cell membrane disruption^70,71^. The current results demonstrated that the MgO NPs combination with CEO resulted in a significantly higher impact compared to MgO NPs or CEO alone, inferring that synergy augmented their influence on cell membrane permeabilization and consequent leakage of cytosolic enzymes.

The purported cell membrane disruption was further confirmed by the significant increase in OD 260 and OD 280 nm absorbing materials, implying leakage of nucleic acids and proteins, respectively, in combination-treated *S. aureus* or *E. coli*, as shown in Fig. 4b and c. It is worth mentioning that although MgO NPs alone did not significantly induce the leakage of nucleic acids in either *S. aureus* or *E. coli*, the combination significantly increased the leakage of nucleic acid by 9- and 10-fold, respectively. Likewise, protein leakage was significantly induced in *E. coli* treated by MgO NPs-CEO despite the insignificant effect of MgO NPs alone.

Metal/metal oxide NPs have been proposed to induce membrane damage by various mechanisms^10,45,50,51,53^. The damage induced may be mechanical due to contact between the rough, rigid multifaceted crystals of MgO NPs and the soft fluid cell membrane of bacterial cells, mostly resulting in abrasion of the cell surface. Conversely, it has been postulated that hydrophobic NPs may penetrate through the lipophilic membrane of bacterial cells, consequently interacting with and inhibiting cytosolic contents such as nucleic acids and proteins^45^.

#### Oxidative stress biomarkers

**Fig. 5:**
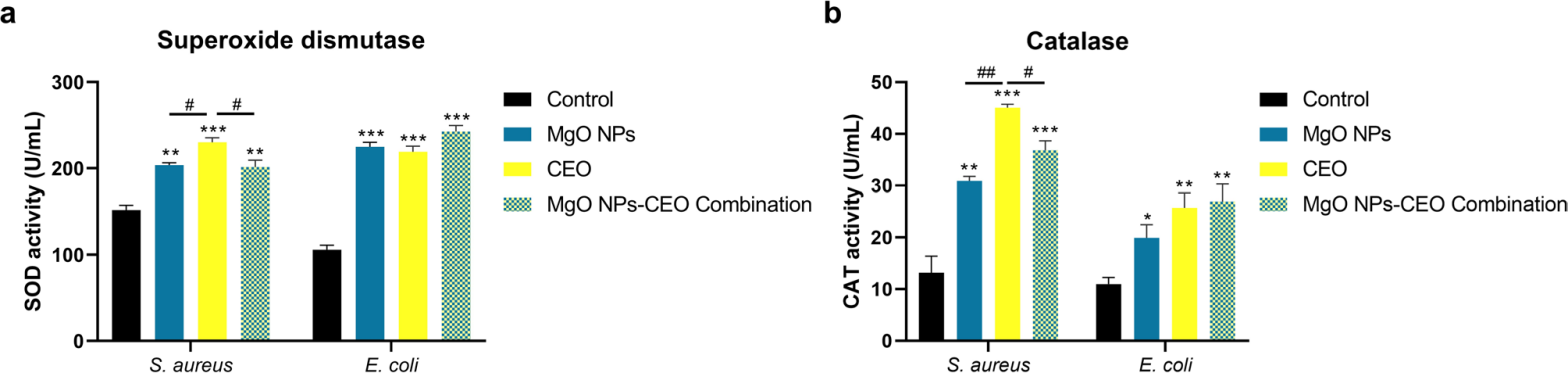
Impact of 1MIC of the phytosynthesized MgO NPs, clove essential oil (CEO) and MgO NPs-CEO combination on the activity of the antioxidant enzymes (**a**) superoxide dismutase (SOD) and (**b**) catalase (CAT) in *S. aureus* (ATCC 29213) and *E. coli* (ATCC 35218). Data are presented as mean ± SD (*n* = 3), and statistical significance was determined by one-way ANOVA test. * designates a significant difference compared to control, whereas # designates a significant difference relative to the specified groups (*/#*p* < 0.05, **/##*p* < 0.01 and ***/###*p* < 0.001).

Antimicrobial agents are known to exert their primary bacteriostatic/bactericidal effects via four major pathways involving cell wall damage, cell membrane disruption and protein and nucleic acid synthesis inhibition. A growing body of evidence reported that antimicrobial agents also exert secondary effects via triggering oxidative stress, which further enhances their primary effect^72,73^. In the current study, the activity of superoxide dismutase (SOD) and catalase (CAT) enzymes was assessed as indicators for the bacterial cell response to the oxidative stress imposed by treatment with MgO NPs, CEO or MgO NPs-CEO combination. As shown in Fig. 5, a significant increase in the activity of the antioxidant enzymes (SOD and CAT) was observed in both *S. aureus* and *E. coli* treated with MgO NPs, CEO and MgO NPs-CEO combination compared to untreated cells.

It is worth mentioning that increased extracellular LDH activity is also related to oxidative stress, since it is implicated in the respiratory metabolism of oxygen and reactive oxygen species generation^74^. In the current study, besides the significant increase in the antioxidant enzyme activity, increased LDH activity in MgO NPs, CEO or MgO NPs-CEO combination-treated cells could also be attributed to its induced intracellular synthesis in response to induced oxidative stress^70^. In bacterial cells, an oxidative stress environment is reported to contribute to changes in membrane permeability and leakage of intracellular components (nucleic acids and proteins)^73^. Thus, in the current study, the primary cell membrane damage imposed by the MgO NPs-CEO combination is further aggravated by the induced oxidative stress. Generally, EOs and/or metal oxide NPs are potential candidates to counter antibiotic resistance, emphasizing that it is not a replacement but rather a way to minimize the use of antibiotics.

## Conclusion

MgO NPs were successfully phytosynthesized employing thyme leaves aqueous extract as an eco-friendly and cost-effective approach. SEM analysis revealed agglomerated spherical NPs with an average size of 55.2 ± 12.8 nm, while EDX confirmed the presence of magnesium and oxygen with elemental compositions of 35.39% and 51.07%, respectively. FTIR analysis elicited characteristic functional groups associated with MgO NPs, while XRD confirmed their cubic crystalline structure. The phytosynthesized MgO NPs and EOs of clove, thyme, sage, and rosemary displayed varying antibacterial activities against *S. aureus*, *E. faecalis*, *E. coli*, and *P. aeruginosa*. The synergistic potential of MgO NPs with the aforementioned EOs was further investigated and combining MgO NPs with clove or thyme EOs exhibited synergistic effects against *S. aureus*,* E. faecalis*, and *E. coli*, whereas MgO NPs combination with sage or rosemary EOs displayed additive and/or indifferent effects against all the tested bacterial strains. Mechanistically, the MgO NPs-CEO combination resulted in a significant bacterial membrane disruption in both *S. aureus* and *E. coli*, as evidenced by significant leakage of intracellular proteins and nucleic acids along with a significant increase in the LDH activity compared to their sole counterparts. Furthermore, this combination also induced oxidative stress, as conveyed by the significant increase in SOD and CAT enzyme activities. Altogether, the current study results highlight the potential of MgO NPs–EO combinations, particularly MgO NPs with CEO, as an effective antibacterial approach.

## Materials and methods

### Materials

Magnesium acetate tetrahydrate was obtained from Alpha Chemika, India. Müeller-Hinton Broth (MHB) was purchased from Oxoid®, UK. Dimethyl sulfoxide (DMSO) was obtained from Fischer Scientific, UK. Lactate dehydrogenase, Catalase and Superoxide Dismutase kits were obtained from Abcam®, UK. All other reagents are of analytical grade.

### Preparation of thyme aqueous extract

Dried leaves of *Thymus vulgaris* were obtained from Imtenan®, Egypt. The leaves were washed with distilled water to remove dust then allowed to air dry in the shade for three days. For thyme aqueous extract preparation, 10 g of thyme leaves were added to 100 mL of deionized water and the mixture was then heated at 80 °C under stirring for 30 min. The extract was then allowed to cool to room temperature and filtered twice through Whatman No.1 filter paper.

### Phytosynthesis of magnesium oxide nanoparticles

For the synthesis of MgO NPs, the aqueous extract of thyme was added gradually to the precursor salt magnesium acetate tetrahydrate solution at a ratio of 1:9 at set temperatures under stirring (250 rpm) for different specified times. The mixture was then set aside for aging overnight. After that, the prepared solid-liquid dispersion was centrifuged at 7000 rpm for 10 min. Subsequently, the precipitate was washed successively with deionized water and ethanol to remove any impurities, then dried overnight at 45 °C. Thereafter, the precipitate was calcinated at 400 °C for 2 h in a muffle furnace. The effects of precursor concentration, temperature and heating time on the phytosynthesis of MgO NPs were investigated and quantified spectrophotometrically according to Table 3.

**Table 3:** Various process conditions for optimization of the green synthesis of MgO NPs.

Constant parameter	Independent variable
Temperature: 60 °C Time: 90 min	Precursor concentration: 0.01 M, 0.1 M and 0.15 M.
Precursor concentration: 0.1 M Time: 90 min	Temperature: 40 °C, 60 °C and 80 °C
Precursor concentration: 0.1 M Temperature: 80 °C	Time: 60 min,90 min and 120 min

### Characterization of the phytosynthesized MgO NPs

The phytosynthesized MgO NPs were preliminarily characterized by a UV spectrophotometer (Thermo Scientific, Evolution 300, USA) at a wavelength ranging from 200 to 600 nm. Morphological features and elemental composition of the MgO NPs were investigated by a scanning electron microscope (SEM) (JSEM-IT 200, JEOL, Ltd., Tokyo, Japan) coupled with an energy-dispersive X-ray analyzer (EDX) unit.

Functional groups present in thyme extract and phytosynthesized MgO NPs were determined using Fourier transform infrared (FTIR) spectra in the scanning range of 450–4000 cm^− 1^. The FTIR spectra of thyme aqueous extract and MgO NPs were assessed by compressing into a disc using dry KCl powder with an IR device (PerkinElmer, USA). The phytosynthesized MgO NPs were also analyzed for crystallinity, purity and size by X-ray diffraction (XRD) (Shimadzu, XRD-7000, Japan).

### Extraction of essential oils

Essential oils were extracted from clove buds, aerial parts of rosemary, sage and thyme (Imtenan^®^, Egypt). The respective plant part was separately subjected to hydro-distillation for 3 h in a Clevenger-type apparatus. The obtained EOs were stored in air-tight glass vials under dark conditions at 4 °C till further use.

### Antibacterial activity

#### Minimum inhibitory concentration (MIC)

The antibacterial activity of the phytosynthesized MgO NPs and EOs of clove, rosemary, sage and thyme was assessed against *Staphylococcus aureus* (ATCC 29213), *Enterococcus faecalis* (ATCC 29122), *Escherichia coli* (ATCC 35218) and *Pseudomonas aeruginosa* (ATCC 27835). The MIC of the respective tested agents was determined by resazurin-based broth microdilution method in accordance with the Clinical and Laboratory Standards Institute (CLSI)^75^. MgO NPs were dispersed in sterile water, while EOs were dissolved in 7.5% v/v DMSO. Serial 2-fold dilutions of MgO NPs and clove, rosemary, sage and thyme EOs were tested in a 96-well microtiter plate containing Müeller-Hinton Broth (MHB) to obtain concentrations ranging from 6 mg/mL to 0.047 mg/mL for MgO NPs and 10 mg/mL to 0.078 mg/mL for clove, rosemary, sage and thyme EOs. Overnight cultures of the tested bacterial strains in MHB were standardized to a 0.5 McFarland turbidity standard. DMSO, uninoculated MHB and inoculated MHB were used as vehicle, sterility and growth controls, respectively. After incubation for 24 h at 35 °C, 30 µL of resazurin (0.015% w/v) was added to all wells, followed by further incubation for 2–4 h. The lowest concentration of the tested agents which did not show any colour change of resazurin was taken as the MIC. Experiments were performed in triplicate.

#### Test for synergism between MgO NPs and EOs

The broth microdilution checkerboard assay was adopted to evaluate the synergistic potential of the phytosynthesized MgO NPs in combination with clove, rosemary, sage, or thyme EOs. As per the predetermined MIC, two-fold dilutions ranging from 1/16 to 4 x MIC of MgO NPs and each EO were added along the x- and y-axis of a 96-well microtiter plate. Afterwards, the respective bacterial inoculum (5 × 10^5^ CFU/mL) was added. The microtiter plate was incubated overnight at 35 °C. Subsequently, resazurin was added to each well and incubated for a further 2**–**4 h. The fractional inhibitory concentration index (FICI) was determined for each double combination in accordance with CLSI^75^. FICI was calculated using the formula FICI = FIC A + FIC B, where FIC A is the MIC of the phytosynthesized MgO NPs in combination with the EO/MIC of the phytosynthesized MgO NPs alone, and FIC B is the MIC of the EO in combination with MgO NPs/MIC of the EO alone. The combination was designated as synergistic when the FICI was ≤ 0.5, additive when the FICI was > 0.5 and ≤ 1.0, indifferent when the FICI was > 1.0 and ≤ 4.0, and antagonistic if the FICI was > 4.

#### Cell membrane integrity assay

The impact of MgO NPs, clove EO (CEO), and MgO NPs–CEO combination on bacterial cell integrity was assessed via nucleic acids and proteins leakage assay^76^. The overnight cultures of *S. aureus* and *E. coli* were adjusted to the 0.5 McFarland turbidity standard, centrifuged at 3000 rpm for 10 min. The pellets left after supernatant discarding were then washed thrice with PBS (pH 7.4). The bacterial strains were then incubated with 1 MIC of MgO NPs, CEO, or MgO NPs-CEO combination for 2 h at 37 °C. Thereafter, treated and untreated samples were centrifuged at 8000 rpm for 5 min and the absorbance of nucleic acids and proteins in the supernatant was assessed at λ_max_ of 260 nm and 280 nm, respectively. The untreated cells (control) were corrected with PBS (pH 7.4), whereas CEO- or MgO NPs-CEO combination-treated samples were corrected using the same concentration of the respective EO alone or in combination with MgO NPs as a blank.

#### Lactate dehydrogenase activity assay

Lactate dehydrogenase (LDH) activity was evaluated as an index for the impact of the phytosynthesized MgO NPs, CEO, and MgO NPs-CEO combination on cell membrane integrity. Overnight cultures of *S. aureus* or *E. coli* adjusted to 0.5 McFarland turbidity standard were centrifuged (3000 rpm for 10 min), washed with PBS and the resuspended pellets were incubated at 37 °C for 2 h with 1 MIC of either MgO NPs, CEO, or MgO NPs-CEO combination. Both treated and untreated bacterial cultures were then centrifuged (8000 rpm for 5 min) and LDH activity in the supernatant of the respective tested bacteria was assessed using an LDH assay kit according to the manufacturer’s instructions^71^.

#### Superoxide dismutase and catalase activity assays

Overnight cultures of *S. aureus* and *E. coli* were centrifuged, washed and resuspended in PBS (pH 7.4). The resuspended pellets were then incubated at 37 °C for 3 h with 1 MIC of MgO NPs, CEO, or MgO NPs-CEO combination. After incubation, the supernatant of the corresponding bacterial strain was assessed for the ability of SOD to inhibit phenazine methosulphate-mediated reduction of nitroblue tetrazolium using the SOD kit as per the manufacturer’s instructions. Catalase activity was also measured spectrophotometrically following the consumption of H_2_O_2_ as per the procedure described in the catalase kit^77^.

### Statistical analysis

Statistical analysis was performed using GraphPad Prism 8 Software (GraphPad Software, Inc.). Statistically significant differences among the groups were determined through one-way ANOVA (analysis of variance) followed by a Tukey post-hoc test.

## Data Availability

All data in this study are publicly available and the raw analysis data can be obtained by contacting the corresponding author upon request.

## References

[1] Tang, K. W. K., Millar, B. C. & Moore, J. E. Antimicrobial resistance (AMR). *Br. J. Biomed. Sci.* **80**, 11387. 10.3389/bjbs.2023.11387 (2023). 37448857 10.3389/bjbs.2023.11387 PMC10336207

[2] Ahmed, S. K. et al. Antimicrobial resistance: impacts, challenges, and future prospects. *J. Med. Surg. Public. Health*. **2**, 100081. 10.1016/j.glmedi.2024.100081 (2024).

[3] Murugaiyan, J. et al. Progress in alternative strategies to combat antimicrobial resistance: focus on antibiotics. *Antibiotics* **11** (2), 200. 10.3390/antibiotics11020200 (2022). 35203804 10.3390/antibiotics11020200 PMC8868457

[4] Mujahid, M. H. et al. Metallic and metal oxide-derived nanohybrid as a tool for biomedical applications. *Biomed. Pharmacother.* **155**, 113791. 10.1016/j.biopha.2022.113791 (2022). 36271568 10.1016/j.biopha.2022.113791

[5] Chavali, M. S. & Nikolova, M. P. Metal oxide nanoparticles and their applications in nanotechnology. *SN Appl. Sci.* **1** (6), 607. 10.1007/s42452-019-0592-3 (2019).

[6] Thakur, N., Ghosh, J., Pandey, S. K., Pabbathi, A. & Das, J. A comprehensive review on biosynthesis of magnesium oxide nanoparticles, and their antimicrobial, anticancer, antioxidant activities as well as toxicity study. *Inorg. Chem. Commun.* **146**, 110156. 10.1016/j.inoche.2022.110156 (2022).

[7] Pathak, J. et al. Exploring the paradigm of phyto-nanofabricated metal oxide nanoparticles: recent advancements, applications, and challenges. *Mol. Biotechnol.* 1–21. 10.1007/s12033-023-00799-8 (2023). 10.1007/s12033-023-00799-8 37436581

[8] Nguyen, N. T. T. et al. A critical review on the bio-mediated green synthesis and multiple applications of magnesium oxide nanoparticles. *Chemosphere* **312**, 137301. 10.1016/j.chemosphere.2022.137301 (2023). 36410506 10.1016/j.chemosphere.2022.137301

[9] Liny, P. et al. Preparation of gold nanoparticles from Helianthus annuus (sun flower) flowers and evaluation of their antimicrobial activities. *Int. J. Pharma Bio Sci.* **3** (1), P–439 (2012).

[10] Muruganandham, M. et al. Tabebuia rosea seed extract mediated synthesis of silver nanoparticles with antibacterial, antioxidant, and antiproliferative activities. *Mater. Res. Express*. **10** (12), 125006. 10.1088/2053-1591/ad1357 (2023).

[11] Lava, M. B., Muddapur, U. M., Basavegowda, N., More, S. S. & More, V. S. Characterization, anticancer, antibacterial, anti-diabetic and anti-inflammatory activities of green synthesized silver nanoparticles using Justica wynaadensis leaves extract. *Mater. Today: Proc.* **46**, 5942–5947. 10.1016/j.matpr.2020.10.048 (2021).

[12] Mishra, K., Basavegowda, N. & Lee, Y. R. Biosynthesis of fe, pd, and Fe–Pd bimetallic nanoparticles and their application as recyclable catalysts for [3 + 2] cycloaddition reaction: a comparative approach. *Catal. Sci. Technol.* **5** (5), 2612–2621. 10.1039/C5CY00099H (2015).

[13] Basavegowda, N., Somu, P., Shabbirahmed, A. M., Gomez, L. A. & Thathapudi, J. J. Bimetallic p-ZnO/n-CuO nanocomposite synthesized using Aegle Marmelos leaf extract exhibits excellent visible-light-driven photocatalytic removal of 4-nitroaniline and Methyl orange. *Photochem. Photobiol. Sci.* **21** (8), 1357–1370. 10.1007/s43630-022-00224-0 (2022). 35451802 10.1007/s43630-022-00224-0

[14] Anicˇić, N., Vukomanović, M., Koklicˇ, T. & Suvorov, D. Fewer defects in the surface slows the hydrolysis rate, decreases the ROS generation potential, and improves the Non-ROS antimicrobial activity of MgO. *Small* **14** (26), 1800205. 10.1002/smll.201800205 (2018). 10.1002/smll.201800205 29782697

[15] Gatou, M. A. et al. Magnesium oxide (MgO) nanoparticles: synthetic strategies and biomedical applications. *Crystals* **14** (3), 215. 10.3390/cryst14030215 (2024).

[16] Rana, A., Yadav, K. & Jagadevan, S. A comprehensive review on green synthesis of nature-inspired metal nanoparticles: mechanism, application and toxicity. *J. Clean. Prod.* **272**, 122880. 10.1016/j.jclepro.2020.122880 (2020).

[17] Vora, L. K. et al. Essential oils for clinical aromatherapy: a comprehensive review. *J. Ethnopharmacol.* 118180. 10.1016/j.jep.2024.118180 (2024). 10.1016/j.jep.2024.118180 38614262

[18] Basavegowda, N., Patra, J. K. & Baek, K. H. Essential oils and mono/bi/tri-metallic nanocomposites as alternative sources of antimicrobial agents to combat multidrug-resistant pathogenic microorganisms: an overview. *Molecules* **25** (5), 1058. 10.3390/molecules25051058 (2020). 32120930 10.3390/molecules25051058 PMC7179174

[19] Basavegowda, N. & Baek, K. H. Synergistic antioxidant and antibacterial advantages of essential oils for food packaging applications. *Biomolecules* **11** (9), 1267. https://www.mdpi.com/2218-273X/11/9/1267 (2021). 10.3390/biom11091267 PMC8466708 34572479

[20] Rotti, R. B. et al. Green synthesis of MgO nanoparticles and its antibacterial properties. *Front. Chem.* **11**, 1143614. 10.3389/fchem.2023.1143614 (2023). 37035117 10.3389/fchem.2023.1143614 PMC10078987

[21] Hirphaye, B. Y., Bonka, N. B., Tura, A. M. & Fanta, G. M. Biosynthesis of magnesium oxide nanoparticles using Hagenia abyssinica female flower aqueous extract for characterization and antibacterial activity. *Appl. Water Sci.* **13** (9), 175. 10.1007/s13201-023-01987-2 (2023).

[22] Jeevanandam, J., Chan, Y. S. & Danquah, M. K. Biosynthesis and characterization of MgO nanoparticles from plant extracts via induced molecular nucleation. *New J. Chem.* **41** (7), 2800–2814. 10.1039/C6NJ03176E (2017).

[23] Essien, E. R., Atasie, V. N., Okeafor, A. O. & Nwude, D. O. Biogenic synthesis of magnesium oxide nanoparticles using manihot esculenta (Crantz) leaf extract. *Int. Nano Lett.* **10** (1), 43–48. 10.1007/s40089-019-00290-w (2020).

[24] Vijayakumar, S., Punitha, V. & Parameswari, N. Phytonanosynthesis of MgO nanoparticles: green synthesis, characterization and antimicrobial evaluation. *Arab. J. Sci. Eng.* **47** (6), 6729–6734. 10.1007/s13369-021-06107-3 (2022).

[25] Younis, I. Y., El-Hawary, S. S., Eldahshan, O. A., Abdel-Aziz, M. M. & Ali, Z. Y. Green synthesis of magnesium nanoparticles mediated from Rosa floribunda charisma extract and its antioxidant, antiaging and antibiofilm activities. *Sci. Rep.* **11** (1), 16868. 10.1038/s41598-021-96377-6 (2021). 34413416 10.1038/s41598-021-96377-6 PMC8376960

[26] Khan, M. I. et al. Green synthesis of magnesium oxide nanoparticles using dalbergia Sissoo extract for photocatalytic activity and antibacterial efficacy. *Appl. Nanosci.* **10** (7), 2351–2364. 10.1007/s13204-020-01414-x (2020).

[27] Rai, A., Singh, A., Ahmad, A. & Sastry, M. Role of halide ions and temperature on the morphology of biologically synthesized gold nanotriangles. *Langmuir* **22** (2), 736–741. 10.1021/la052055q (2006). 16401125 10.1021/la052055q

[28] Radulescu, D. M. et al. Green synthesis of metal and metal oxide nanoparticles: a review of the principles and biomedical applications. *Int. J. Mol. Sci.* **24** (20), 15397. 10.3390/ijms242015397 (2023). 37895077 10.3390/ijms242015397 PMC10607471

[29] Alighourchi, H. & Barzegar, M. Some physicochemical characteristics and degradation kinetic of anthocyanin of reconstituted pomegranate juice during storage. *J. Food Eng.* **90** (2), 179–185. 10.1016/j.jfoodeng.2008.06.019 (2009).

[30] Bandeira, M., Giovanela, M., Roesch-Ely, M., Devine, D. M. & da Silva Crespo, J. Green synthesis of zinc oxide nanoparticles: A review of the synthesis methodology and mechanism of formation. *Sustainable Chem. Pharm.* **15**, 100223. 10.1016/j.scp.2020.100223 (2020).

[31] Shawky, A. M. & El-Tohamy, M. F. Highly functionalized modified metal oxides polymeric sensors for potentiometric determination of letrozole in commercial oral tablets and biosamples. *Polymers* **13** (9), 1384. 10.3390/polym13091384 (2021). 33922800 10.3390/polym13091384 PMC8123036

[32] Sharma, G., Soni, R. & Jasuja, N. D. Phytoassisted synthesis of magnesium oxide nanoparticles with swertia Chirayaita. *J. Taibah Univ. Sci.* **11** (3), 471–477. 10.1016/j.jtusci.2016.09.004 (2017).

[33] Nguyen, D. T. C. et al. Biogenic synthesis of MgO nanoparticles from different extracts (flower, bark, leaf) of Tecoma stans (L.) and their utilization in selected organic dyes treatment. *J. Hazard. Mater.* **404**, 124146. 10.1016/j.jhazmat.2020.124146 (2021). 33053473 10.1016/j.jhazmat.2020.124146

[34] Balashanmugam, P. & Kalaichelvan, P. T. Biosynthesis characterization of silver nanoparticles using Cassia Roxburghii DC. aqueous extract, and coated on cotton cloth for effective antibacterial activity. *Int. J. Nanomed.* **10** (sup2), 87–97. 10.2147/IJN.S79984 (2015). 10.2147/IJN.S79984 PMC4599608 26491310

[35] Pathania, D. et al. Essential oil-mediated biocompatible magnesium nanoparticles with enhanced antibacterial, antifungal, and photocatalytic efficacies. *Sci. Rep.* **12** (1), 11431. 10.1038/s41598-022-14984-3 (2022). 35794190 10.1038/s41598-022-14984-3 PMC9259627

[36] Oves, M. et al. Green synthesis of silver nanoparticles by Conocarpus lancifolius plant extract and their antimicrobial and anticancer activities. *Saudi J. Biol. Sci.* **29** (1), 460–471. 10.1016/j.sjbs.2021.09.007 (2022). 35002442 10.1016/j.sjbs.2021.09.007 PMC8716933

[37] Muhaymin, A. et al. Green synthesis of magnesium oxide nanoparticles using hyphaene Thebaica extract and their photocatalytic activities. *Sci. Rep.* **14** (1), 20135. 10.1038/s41598-024-71149-0 (2024). 39210024 10.1038/s41598-024-71149-0 PMC11362519

[38] Rodríguez-Hernández, A. P., Vega-Jiménez, A. L., Vázquez-Olmos, A. R., Ortega-Maldonado, M. & Ximenez-Fyvie, L. A. Antibacterial properties in vitro of magnesium oxide nanoparticles for dental applications. *Nanomaterials* **13** (3), 502. 10.3390/nano13030502 (2023). 36770464 10.3390/nano13030502 PMC9921384

[39] Bhattacharya, P., Dey, A. & Neogi, S. An insight into the mechanism of antibacterial activity by magnesium oxide nanoparticles. *J. Mater. Chem. B*. **9** (26), 5329–5339. 10.1039/D1TB00875G (2021). 34143165 10.1039/d1tb00875g

[40] Proniewicz, E., Vijayan, A. M., Surma, O., Szkudlarek, A. & Molenda, M. Plant-Assisted green synthesis of MgO nanoparticles as a sustainable material for bone regeneration: spectroscopic properties. *Int. J. Mol. Sci.* **25** (8), 4242. 10.3390/ijms25084242 (2024). 38673825 10.3390/ijms25084242 PMC11050608

[41] Dehkordi, P. H., Brujeni, H. M. & Abbasvali, M. Evaluation of the inhibitory effects of magnesium oxide and copper oxide nanoparticles on biofilm formation of some foodborne bacterial pathogens. *J. Nanosci. Nanotechnol Appl.* **6**, 104 (2022).

[42] Nguyen, N. Y. T., Grelling, N., Wetteland, C. L., Rosario, R. & Liu, H. Antimicrobial activities and mechanisms of magnesium oxide nanoparticles (nMgO) against pathogenic bacteria, yeasts, and biofilms. *Sci. Rep.* **8** (1), 16260. 10.1038/s41598-018-34567-5 (2018). 30389984 10.1038/s41598-018-34567-5 PMC6214931

[43] Krishnamoorthy, K., Manivannan, G., Kim, S. J., Jeyasubramanian, K. & Premanathan, M. Antibacterial activity of MgO nanoparticles based on lipid peroxidation by oxygen vacancy. *J. Nanopart. Res.* **14**, 1–10. 10.1007/s11051-012-1063-6 (2012).22448125

[44] Jayakumar, S. et al. Antibacterial effectiveness of zinc oxide and magnesium oxide nanoparticles against *Enterococcus faecalis*: an in vitro study. *J. Int. Oral Health* **17** (2). https://journals.lww.com/jioh/fulltext/2025/03000/antibacterial_effectiveness_of_zinc_oxide_and.2.aspx (2025).

[45] Kadiyala, U., Kotov, N. A. & VanEpps, J. S. Antibacterial metal oxide nanoparticles: challenges in interpreting the literature. *Curr. Pharm. Design*. **24** (8), 896–903. 10.2174/1381612824666180219130659 (2018). 10.2174/1381612824666180219130659 PMC5959755 29468956

[46] Franco, D., Calabrese, G., Guglielmino, S. P. P. & Conoci, S. Metal-based nanoparticles: antibacterial mechanisms and biomedical application. *Microorganisms* **10** (9), 1778. 10.3390/microorganisms10091778 (2022). 36144380 10.3390/microorganisms10091778 PMC9503339

[47] Cha, S. H. et al. Shape-Dependent biomimetic Inhibition of enzyme by nanoparticles and their antibacterial activity. *ACS Nano*. **9** (9), 9097–9105. 10.1021/acsnano.5b03247 (2015). 26325486 10.1021/acsnano.5b03247

[48] Moore, T. L. et al. Petri-Fink, nanoparticle colloidal stability in cell culture media and impact on cellular interactions. *Chem. Soc. Rev.* **44** (17), 6287–6305. 10.1039/C4CS00487F (2015). 26056687 10.1039/c4cs00487f

[49] Akshaykranth, A., Jayarambabu, N., Tumu, V. R. & Rajaboina, R. K. Comparative study on antibacterial activity of MgO nanoparticles synthesized from lawsonia inermis leaves extract and chemical methods. *J. Inorg. Organomet. Polym Mater.* **31** (6), 2393–2400. 10.1007/s10904-021-01915-4 (2021).

[50] Tamilselvi, R. et al. Antimicrobial activity of metal oxide nanoparticles. *Biomedical Pharmacol. J.* **17** (3), 1757–1767. 10.13005/bpj/2981 (2024).

[51] Azam, A. et al. Antimicrobial activity of metal oxide nanoparticles against gram-positive and gram-negative bacteria: a comparative study. *Int. J. Nanomed.* **7** (null) 6003–6009. 10.2147/IJN.S35347 (2012). 10.2147/IJN.S35347 PMC3519005 23233805

[52] Stoimenov, P. K., Klinger, R. L., Marchin, G. L. & Klabunde, K. J. Metal oxide nanoparticles as bactericidal agents. *Langmuir* **18** (17), 6679–6686. 10.1021/la0202374 (2002).

[53] Nachimuthu, S. et al. Facile synthesis of ZnO-Y2O3 nanocomposite for photocatalytic and biological applications. *Catal Commun.* **184**, 106786. 10.1016/j.catcom.2023.106786 (2023).

[54] Russell, A. Similarities and differences in the responses of microorganisms to biocides. *J. Antimicrob. Chemother.* **52** (5), 750–763. 10.1093/jac/dkg422 (2003). 14519671 10.1093/jac/dkg422

[55] Yi, H. et al. Drug combinations to prevent antimicrobial resistance: various correlations and laws, and their verifications, thus proposing some principles and a preliminary scheme. *Antibiotics* **11** (10), 1279. 10.3390/antibiotics11101279 (2022). 36289938 10.3390/antibiotics11101279 PMC9598766

[56] Angane, M., Swift, S., Huang, K., Butts, C. A. & Quek, S. Y. Essential oils and their major components: an updated review on antimicrobial activities, mechanism of action and their potential application in the food industry. *Foods* **11** (3), 464. 10.3390/foods11030464 (2022). 35159614 10.3390/foods11030464 PMC8833992

[57] Galovičová, L. et al. Thymus vulgaris essential oil and its biological activity. *Plants* **10** (9), 1959. 10.3390/plants10091959 (2021). 34579491 10.3390/plants10091959 PMC8467294

[58] Haro-González, J. N., Castillo-Herrera, G. A., Martínez-Velázquez, M. & Espinosa-Andrews, H. Clove essential oil (Syzygium aromaticum L. Myrtaceae): extraction, chemical composition, food applications, and essential bioactivity for human health. *Molecules* **26** (21), 6387. 10.3390/molecules26216387 (2021). 34770801 10.3390/molecules26216387 PMC8588428

[59] Craft, J. D., Satyal, P. & Setzer, W. N. The chemotaxonomy of common Sage (Salvia officinalis) based on the volatile constituents. *Medicines* **4** (3), 47. 10.3390/medicines4030047 (2017). 28930262 10.3390/medicines4030047 PMC5622382

[60] Jiang, Y. et al. Chemical composition and antimicrobial activity of the essential oil of Rosemary. *Environ. Toxicol. Pharmacol.* **32** (1), 63–68. 10.1016/j.etap.2011.03.011 (2011). 21787731 10.1016/j.etap.2011.03.011

[61] Khwaza, V. & Aderibigbe, B. A. Antibacterial activity of selected essential oil components and their derivatives: A review. *Antibiotics* **14** (1), 68. 10.3390/antibiotics14010068 (2025). 39858354 10.3390/antibiotics14010068 PMC11761885

[62] El-Hosseiny, L., El-Shenawy, M., Haroun, M. & Abdullah, F. Comparative evaluation of the inhibitory effect of some essential oils with antibiotics against Pseudomonas aeruginosa. *Int. J. Antibiot.* **2014** (1), 586252. 10.1155/2014/586252 (2014).

[63] López-Cano, A. A. et al. Chemically modified nanoparticles for enhanced antioxidant and antimicrobial properties with cinnamon essential oil. *Antioxidants*10.3390/antiox12122057 (2023). 38136177 10.3390/antiox12122057 PMC10740917

[64] Oroojalian, F., Orafaee, H. & Azizi, M. Synergistic antibaterial activity of medicinal plants essential oils with biogenic silver nanoparticles. *Nanomed. J.* **4** (4), 237–244. 10.22038/nmj.2017.04.006 (2017).

[65] Mussin, J. & Giusiano, G. Synergistic antimicrobial activity of biogenic silver nanoparticles and acanthospermum australe essential oil against skin infection pathogens, antibiotics. 10.3390/antibiotics13070674 (2024). 10.3390/antibiotics13070674 PMC11274195 39061356

[66] Heydari, M. A., Mobini, M. & Salehi, M. The synergic activity of Eucalyptus leaf oil and silver nanoparticles against some pathogenic bacteria. *Arch. Pediatr. Infect. Dis.* **5** (4), e61654. 10.5812/pedinfect.61654 (2017).

[67] Manju, S. et al. Antibacterial, antibiofilm and cytotoxic effects of Nigella sativa essential oil coated gold nanoparticles. *Microb. Pathog.* **91**, 129–135. 10.1016/j.micpath.2015.11.021 (2016). 26703114 10.1016/j.micpath.2015.11.021

[68] Morsy, M. K., Khalaf, H. H., Sharoba, A. M., El-Tanahi, H. H. & Cutter, C. N. Incorporation of essential oils and nanoparticles in Pullulan films to control foodborne pathogens on meat and poultry products. *J. Food Sci.* **79** (4), M675–M684. 10.1111/1750-3841.12400 (2014). 24621108 10.1111/1750-3841.12400

[69] Yadav, Y., Dinesh, A. K., Kumari, M. & Maheshwari, R. K. Ethnopharmacology and traditional attributes of clove (Syzygium aromaticum). *Int. J. Environ. Health Sci.* **4** (1), 35–38. 10.47062/1190.0401.05 (2022).

[70] Lin, Y., Wang, Y. & Li, P. Mutual regulation of lactate dehydrogenase and redox robustness. *Front. Physiol.* **13**, 1038421. 10.3389/fphys.2022.1038421 (2022). 36407005 10.3389/fphys.2022.1038421 PMC9672381

[71] Castro-Valenzuela, B. E. et al. Rodríguez-Padilla, antibacterial efficacy of novel bismuth-silver nanoparticles synthesis on *Staphylococcus aureus* and *Escherichia coli* infection models. *Front. Microbiol.* **15**10.3389/fmicb.2024.1376669 (2024). 10.3389/fmicb.2024.1376669 PMC11033500 38650875

[72] Li, H. et al. Reactive oxygen species in pathogen clearance: the killing mechanisms, the adaption response, and the side effects. *Front. Microbiol.* **11**10.3389/fmicb.2020.622534 (2021). 10.3389/fmicb.2020.622534 PMC7889972 33613470

[73] Kong, A. S. Y. et al. Lai, Anti- and Pro-Oxidant properties of essential oils against antimicrobial resistance. *Antioxidants* **11** (9), 1819. 10.3390/antiox11091819 (2022). 36139893 10.3390/antiox11091819 PMC9495521

[74] Jovanovic, P. et al. Lactate dehydrogenase and oxidative stress activity in primary open-angle glaucoma aqueous humour. *Bosnian J. Basic. Med. Sci.* **10** (1), 83–88. 10.17305/bjbms.2010.2743 (2010). 10.17305/bjbms.2010.2743 PMC5596618 20192938

[75] CLSI. Performance standards for antimicrobial susceptibility testing Clinical and Laboratory Standards Institute, Wayne, PA. (2019).

[76] Wijesundara, N. M., Lee, S. F., Cheng, Z., Davidson, R. & Rupasinghe, H. P. V. Carvacrol exhibits rapid bactericidal activity against Streptococcus pyogenes through cell membrane damage. *Sci. Rep.* **11** (1), 1487. 10.1038/s41598-020-79713-0 (2021). 33452275 10.1038/s41598-020-79713-0 PMC7811018

[77] Piella, J., Bastús, N. G. & Puntes, V. Modeling the optical responses of noble metal nanoparticles subjected to physicochemical transformations in physiological environments: aggregation, dissolution and oxidation. *Z. FÃ¼r Phys. Chem.* **231** (1), 33–50. 10.1515/zpch-2016-0874 (2017).

